# White matter organisation of sensorimotor tracts is associated with motor imagery in childhood

**DOI:** 10.1007/s00429-024-02813-4

**Published:** 2024-06-25

**Authors:** Mugdha Mukherjee, Christian Hyde, Pamela Barhoun, Kaila M Bianco, Mervyn Singh, Jessica Waugh, Timothy J Silk, Jarrad AG Lum, Karen Caeyenberghs, Jacqueline Williams, Peter G Enticott, Ian Fuelscher

**Affiliations:** 1 School of Psychology, Deakin University, 221 Burwood Hwy, Burwood VIC 3125, Geelong, VIC Australia; 2 Institute for Health and Sport, Victoria University, Melbourne, VIC Australia

**Keywords:** Motor imagery, White matter, Fixel-based analysis, Cerebellar peduncles, Childhood

## Abstract

Despite the important role of motor imagery (MI) in motor development, our understanding of the contribution of white matter fibre properties to MI performance in childhood remains limited. To provide novel insight into the white matter correlates of MI performance, this study examined the association between white matter fibre properties and motor imagery performance in a sample of typically developing children. High angular diffusion weighted imaging data were collected from 22 typically developing children aged 6–14 years (12 female, *M*_Age_= 10.56). Implicit motor imagery performance was assessed using a mental hand rotation paradigm. The cerebellar peduncles and the superior longitudinal fasciculus were reconstructed using TractSeg, a semi-automated method. For each tract, white matter microstructure (fibre density, FD) and morphology (fibre bundle cross-section, FC) were estimated using Fixel-Based Analysis. Permutation-based inference testing and partial correlation analyses demonstrated that higher FC in the middle cerebellar peduncles was associated with better MI performance. Tract-based region of interest analyses showed that higher FC in the middle and superior cerebellar peduncles were associated with better MI performance. Results suggest that white matter connectivity along the cerebellar peduncles may facilitate MI performance in childhood. These findings advance our understanding of the neurobiological systems that underlie MI performance in childhood and provide early evidence for the relevance of white matter sensorimotor pathways to internal action representations.

**Supplementary Information:**

The online version contains supplementary material available at 10.1007/s00429-024-02813-4.

## Introduction

Motor Imagery (MI), the ability to mentally simulate an action without engaging in overt movement (Decety 1996), plays a vital role in childhood motor development. In support of this view, studies suggest that the ability to perform MI in childhood is associated with the development and expression of important motor functions, including motor planning (Fuelscher et al. 2016; Toussaint et al. 2013) and adaptive control (Fuelscher et al. 2015b). Further, where motor skill is impaired in childhood (e.g., in children with developmental coordination disorder), MI performance is often atypical (Barhoun et al. 2019; Reynolds et al. 2015; Williams et al. 2008). Together, these converging lines of evidence suggest that the ability to internally simulate movement (or the ability to perform MI) may be associated with motor outcomes in childhood.

From a neurocomputational perspective, recent models of human motor control suggest that MI performance provides insight into the ability to generate and/or engage internal models of action. These internal models (also referred to as forward models; Miall and Wolpert 1996) assist in predicting the sensory consequences of movement and thus contribute to a flexible and refined motor system (Franklin & Wolpert, 2011; Ishikawa et al. 2016). In support of the MI framework, research suggests that overt and imagined movements share similar temporal and biomechanical characteristics (Kilteni et al. 2018). Complementing this work, studies demonstrate close correspondence in the brain regions involved during overt and imagined movement, including the prefrontal, premotor and parietal cortices, the supplementary motor area, the basal ganglia and the cerebellum (Hardwick et al. 2018; Hétu et al. 2013; Zapparoli et al. 2014). Accordingly, MI paradigms are considered to provide insight into the internal action representations that unconsciously precede and subserve movement (Gabbard 2009; Munzert et al. 2009).

The literature commonly distinguishes between implicit and explicit MI (McAvinue and Robertson 2008). During explicit MI tasks, participants are actively instructed to imagine a given movement. Implicit MI tasks, on the other hand, elicit the use of MI without specific instructions to engage in MI (Hanakawa 2016). Possible advantages of implicit MI paradigms are that task performance is less susceptible to cognitive penetrability (Pylyshyn 2002) and the effects of introspection. Further, unlike the majority of explicit motor imagery tasks, the use of MI can be objectively inferred based on participants’ performance profiles (de Lange et al. 2006; Parsons 1994; Sekiyama et al. 2014; ter Horst et al. 2010). Accordingly, implicit MI paradigms are commonly adopted in childhood settings to measure the unconscious processes that subserve movement.

A widely used task to measure implicit MI in childhood is the hand rotation task (HRT; Parsons 1987). This implicit MI task requires participants to identify the laterality of hand stimuli presented at various angular rotations, and in different postural views. Studies adopting the HRT have shown that MI develops in a non-linear fashion, with substantial performance increases observed between the ages of 6–12 years and more subtle improvements observed thereafter (Caeyenberghs et al. 2009; Fuelscher et al. 2015b; Souto et al. 2020). Since comparable non-linear trajectories have also been observed for the development of motor planning (Fuelscher et al. 2016; Wilmut and Byrne 2014) and adaptive control (Fuelscher et al. 2015b; Wilson and Hyde 2013), this research has contributed to a growing body of work demonstrating the relevance of MI to motor development. Still, despite the important insights that MI provides into childhood motor function, our understanding of the neurobiological mechanisms associated with MI performance remains limited. This represents an important knowledge gap since an improved understanding of these mechanisms can assist in the identification of neurobiological markers of typical development and inform the design and evaluation of MI training programs that are now commonly adopted to support motor development in childhood (Scott et al. 2021; Wilson et al. 2016).

What is currently known about the neurobiological basis of implicit MI is largely derived from task-based functional MRI studies examining patterns of brain activation while participants perform the HRT (Hardwick et al. 2018; Hétu et al. 2013; Zapparoli et al. 2014). These studies have highlighted several brain regions that show an increased blood-oxygen-level-dependent (BOLD) response during MI. These include frontal motor regions (the inferior, middle, and superior frontal gyri and the supplementary motor area), parietal regions (the inferior and superior parietal lobes and the postcentral gyrus), and the cerebellum (lobules VI and VII; Hardwick et al. 2018; Hétu et al. 2013; Zapparoli et al. 2014). Although this research has provided valuable insight into the brain regions associated with implicit MI performance, these results were based on adult studies. As a result, little is known about the relevance of these regions to MI in childhood. Further, while several studies have considered the functional relevance of individual differences in grey matter to MI performance, the degree to which individual differences in white matter organisation may contribute to MI in childhood remains to be investigated. This is surprising since white matter pathways connect many of the cortical regions (Mori et al. 2005) considered to be involved during MI and given the importance of white matter organisation to childhood motor function (Bhoyroo et al. 2022; Fuelscher et al. 2021; Grohs et al. 2018).

White matter pathways consist of myelinated axons that facilitate information transmission between distant brain regions (Geeraert et al. 2020). Two sensorimotor white matter pathways that present as leading candidates for subserving MI in childhood are the superior longitudinal fasciculus (SLF) and the cerebellar peduncles. In support of this view, adult studies suggest that these pathways connect sensorimotor grey matter regions that play a role during MI, including frontal motor regions, parietal regions and the cerebellum (Hardwick et al. 2018; Hétu et al. 2013; Zapparoli et al. 2014). Indeed, the SLF acts as a principal connection between fronto-dorsal (including the premotor and supplementary motor areas) and parietal cortices (Kamali et al. 2014) while the cerebellar peduncles connect the cerebellum to various sensorimotor regions (premotor cortex, primary motor cortex, posterior parietal cortex, and basal ganglia; Bostan and Strick 2010; Cacciola et al. 2017; Welniarz et al. 2021).

To provide novel insight into the neurostructural basis of MI, the aim of this study was to assess the degree to which white matter organisation of the SLF and the cerebellar peduncles were associated with MI performance in a sample of typically developing children. The SLF and the cerebellar peduncles were chosen as tracts of interest in this study since they connect many of the cortical regions considered to be involved during MI and given their documented role in childhood motor development. MI performance was assessed using the HRT. White matter fibre properties were assessed using fixel-based analysis (FBA; Raffelt et al. 2017), a novel and fibre-specific analysis framework that offers increased specificity and biological interpretability relative to traditional (voxel-based) approaches. It was hypothesised that white matter organisation of the SLF and the cerebellar peduncles would be positively associated with MI performance.

## Methods

### Participants

Participants included 22 healthy children aged 6–14 years (12 female; four left-handed; *M*_Age_= 10.56; *SD*_Age_= 2.05) who were recruited through university advertisements and social media. Parents of participants provided written informed consent and parents of participating children were reimbursed for their time. Exclusion criteria included a prior diagnosis (collected using a parent questionnaire) of a medical or neurodevelopmental condition (e.g., developmental coordination disorder, attention deficit hyperactivity disorder, autism spectrum disorder) or an intellectual disability. All procedures were performed in compliance with relevant laws and institutional guidelines and have been approved by the relevant institutional ethics committee (Ref no. 2019-009; 2018-037).

### Hand rotation task

**Fig. 1:**
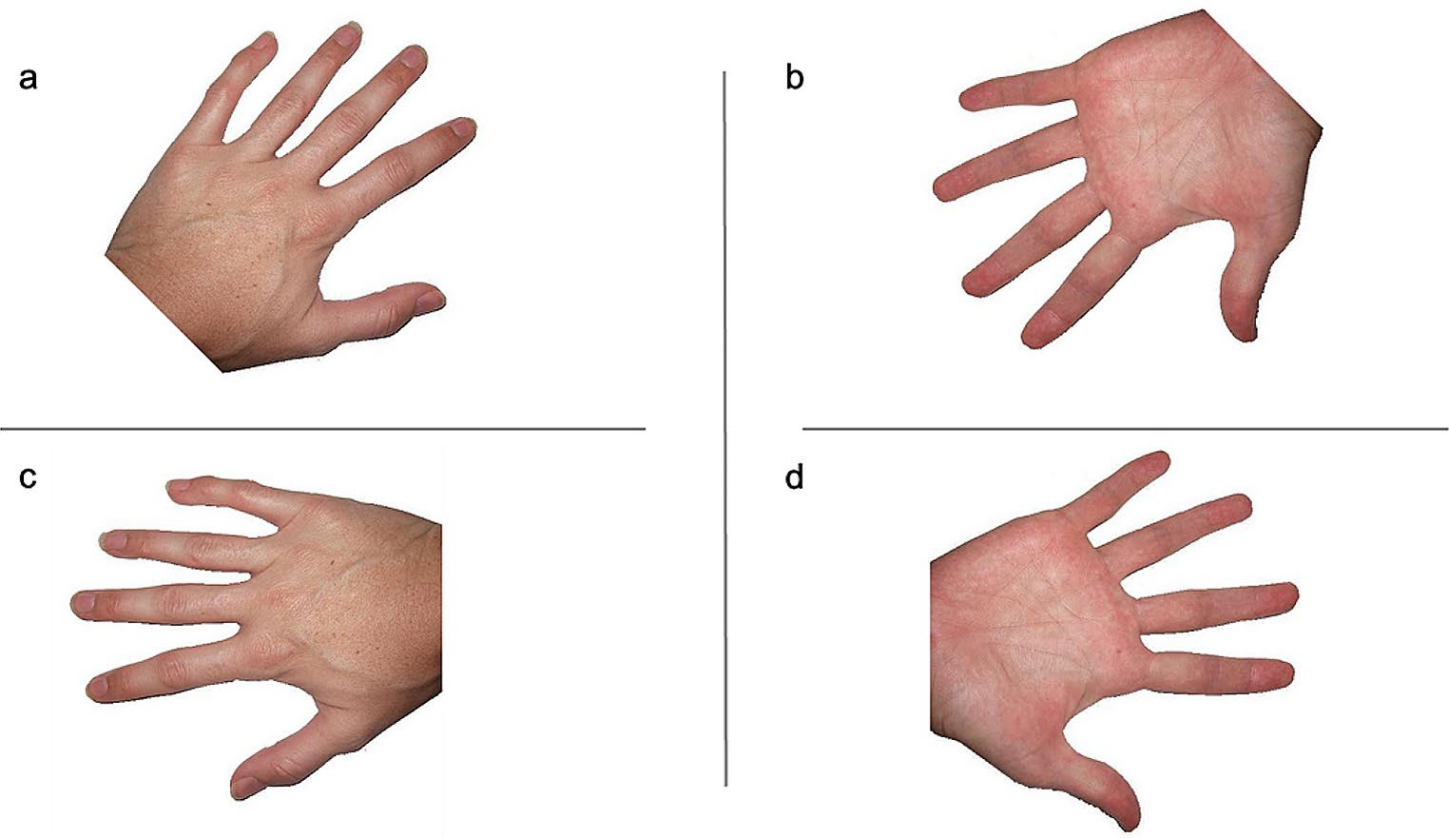
Hand rotation task stimuli. a: left-hand stimulus rotated 45$^\circ$ (medial) in back view; b: left-hand stimulus rotated 225$^\circ$ (lateral) in palm view; c: right-hand stimulus rotated 270° (medial) in back view; d: right-hand stimulus rotated 90° (lateral) in palm view

MI performance was assessed using the HRT (Parsons 1994). The task was programmed using E-Prime software (Psychology Software Tools, Pittsburgh, PA, USA). Participants were presented with single hand stimuli (9 cm × 8 cm, centred in the middle of the screen) on a laptop computer. They were seated upright with their left index fingers placed on the ‘D’ key of the keyboard (designated for left hands) and their right index fingers on the ‘K’ key (designated for right hands). The back of participants’ hands was visible to them as they completed the task. During the task, participants were required to decide as quickly and accurately as possible whether each presented stimulus was a left or right hand. No specific instructions cueing MI were provided. The hand stimuli were presented either in back view (back of the hand facing the participant) or palm view (palm of the hand facing the participant) and were presented randomly in 45° increments between 0° and 360° (see Fig. 1 for example stimuli). To ensure that participants did not use a visual matching strategy to perform the task, participants were instructed to keep their hands on the keyboard while they performed the task.

The hand stimuli remained on the screen until the participant made a response by pressing the designated keys on the keyboard of the laptop or until 10 s had passed. Participants completed five practice trials followed by 40 test trials. For statistical analysis, trials were combined across mirror equivalent angels (e.g., 90° was combined with 270°) to obtain eight trials per angular rotation (0°, 45°, 90°, 135°, 180°). Response time (RT) to the nearest 1 ms and accuracy for each hand stimulus were recorded. Trials with response times below 250 ms were removed to account for anticipatory responses (Hyde et al. 2013, 2014; Kosslyn et al. 1998). This resulted in the removal of one trial each from two participants. Similar to previous work (Butson et al. 2014; Fuelscher et al. 2016), a minimum accuracy criterion of 60% correct responses for hands presented at 0°/360° in the back view was applied to ensure that participants were able to differentiate hand laterality at the most basic level of stimulus presentation. This resulted in the removal of two participants from the analysis. Two further participants were excluded from the analysis due to missing HRT data. Thus, analyses involving HRT data were based on a final sample of *N* = 18.

Mean RT and accuracy for each hand stimulus in both postural views and at each angle of rotation was calculated for every participant. Performance was averaged across angular rotation of 0°, 45°, 90°, 135°, and 180°. Mean accuracy was calculated as the proportion of correct responses across all angles. As per previous work examining HRT performance in children and young adults (Barhoun et al. 2019; Fuelscher et al. 2015a, b; Hyde et al. 2014, 2018), we calculated a mean inverse efficiency score (IES) for each participant by dividing the mean response time across all trials by the proportion of correct responses at each of the stimuli presentation conditions (thus lower IES values indicate better task performance). Medial rotation performance was calculated as the average of responses for left hands presented at 45°, 90°, and 135° and right hands presented at 315°, 270°, and 225°. Lateral rotation performance was calculated as the average responses for left hands presented at 315°, 270°, and 225° and right hands presented at 45°, 90° and 135°.

To establish the use of a MI strategy when completing the HRT (as opposed to a visual imagery strategy; see Mibu et al. 2020), participants’ performance profiles were examined individually to ensure their responses were consistent with the biomechanical constraints of movement. This is considered a unique feature to implicit MI performance (Butson et al. 2014; Kosslyn et al. 1998; Spruijt, van der Kamp, et al., 2015b; ter Horst et al. 2010) where performance on biomechanically complex rotations (lateral rotations) are expected to be less efficient compared to the biomechanically simpler rotations (medial rotations). Similar to previous work (Barhoun et al. 2021; Hyde et al. 2018), participants’ use of a MI strategy was inferred when their absolute mean efficiency on lateral rotations exceeded their absolute mean efficiency value on medial rotations. All participants included in the final analysis met this criterion and were therefore considered to have engaged in a MI strategy during the task.

### Neuroimaging

#### MRI acquisition

MR scanning was conducted using a Siemens MAGNETOM Prisma 3T scanner with a total scanning time of approximately 30 min. The scan was administered by professional radiographers who had extensive experience conducting MRI in children. To reduce motion during the scan, we adopted a child-focused approach and individualised familiarisation strategies. These included a training session in a mock scanner to help participants acclimate to the MRI environment. During the scan, participants lay supine on the scanner bed and watched a video of their choice. The radiographer monitored participants verbally and visually during the scan. Where the radiographer observed increased motion, participants were reminded to lay still, or the sequence was repeated if time allowed.

High resolution T1-weighted, 3D MPRAGE images were acquired for each participant in the sagittal plane, using the following parameters: TR = 1900 ms, TI = 900 ms, TE = 2.49 ms, flip angle = 9$^\circ$, voxel size = 0.9 mm^3^, FoV = 240 mm, 192 contiguous slices with an acquisition time of 4:26 min. High-angular resolution data were acquired in the transverse plane with an anterior-posterior phase encoding direction (PE). We acquired 64 gradient directions (b = 3000 s/mm2, 8 interleaved b = 0 volumes) using the following parameters: TR = 8400 ms, TE = 110 ms, flip angle = 90°, voxel size = 2.5 mm^3^, FoV = 240 mm, multi-band factor = 2. A pair of reverse phase-encoded *b* = 0 images were also acquired to correct for magnetic susceptibility-induced distortions during EPI acquisition. The total acquisition time of the diffusion sequence was 11:46 min.

### Pre-processing

Data were pre-processed using MRtrix3 (Tournier et al. 2019) and MRtrix3Tissue (https://3Tissue.github.io) a fork of MRtrix3. Pre-processing steps included denoising (Veraart et al. 2016), Gibbs ringing removal (Kellner et al. 2016), between volume motion and eddy current distortion correction with outlier replacement (Andersson et al. 2016; Bastiani et al. 2019; see supplementary Table S4 for motion estimates), susceptibility induced (EPI) distortion (Andersson et al. 2003), and bias field correction (Tustison et al. 2010). Following pre-processing, data were upsampled to an isotropic voxel size of 1.25mm^3^. These pre-processing steps were applied to all data except four participants. For two of these participants, susceptibility-induced distortion correction was not applied as reverse-phase encoded images were not available. For the remaining two participants, bias field correction was not applied as this step resulted in poor brain mask estimation. Since these steps are considered optional in the recommended FBA analysis pipeline (Tournier et al. 2019; Dhollander et al. 2021), these participants were included in the final analysis.

### Fiber orientation distribution and fixel metric calculations

Response functions for white matter, grey matter, and cerebrospinal fluid were estimated for each participant and averaged across participants to generate group level response functions for each tissue type. We then performed Single-Shell 3-Tissue CSD (SS3T-CSD) for each participant using the group average response functions to generate individual fiber orientation distribution (FOD) maps (Dhollander and Connelly 2016). Following intensity normalization (Raffelt et al. 2017), a population template specific to the study was generated using the FOD maps from all 22 participants. Individual FOD maps were subsequently registered to the population template and segmented to generate individual fixel maps for each participant (Raffelt et al. 2017).

For each participant, measures of fibre density (a microscopic measure of axonal density or packing; Raffelt et al. 2017) and fibre bundle cross-section (a morphological estimate of the macroscopic difference in fibre bundle diameter or size; Raffelt et al. 2017) were derived at every white matter fixel in the brain, as described in Raffelt et al. (2017). This resulted in whole-brain fibre density (FD) and fibre bundle cross-section (FC) fixel maps for each participant. As per the recommended analysis pipeline (Tournier et al. 2019; Dhollander et al. 2021), FC was log transformed prior to statistical analysis.

### Tractography

**Fig. 2:**
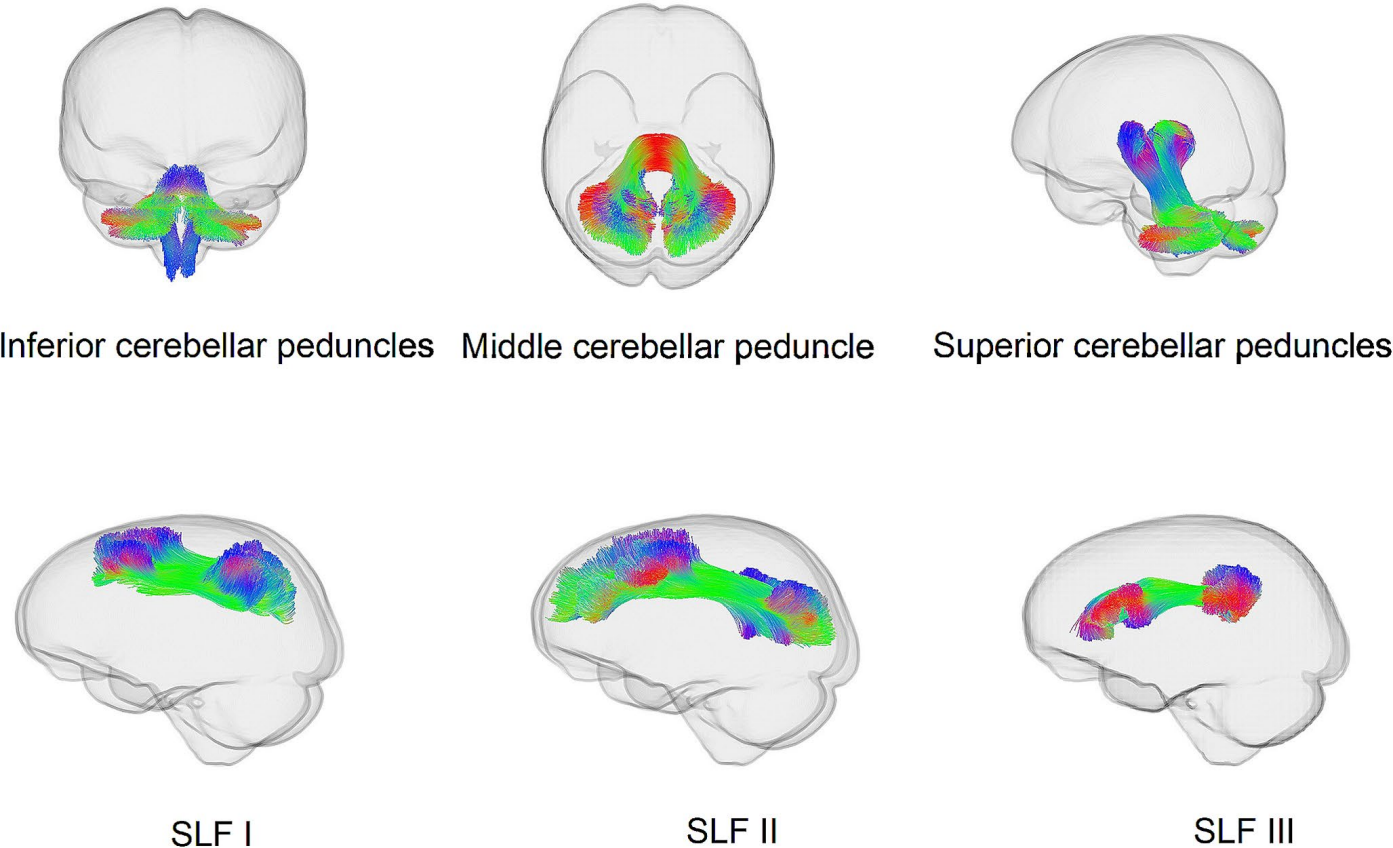
Glass brains depicting the cerebellar peduncles and the SLFs, delineated using TractSeg (Wasserthal et al. 2018). TractSeg was applied to the study specific population template. For visualisation purposes, the left and right hemispheres were combined to generate a single bilateral tractogram for each tract. Colours represent streamline directions; red: left–right, green: anterior–posterior, blue: inferior–superior

TractSeg (Wasserthal et al. 2018, 2019), a semi-automated probabilistic tractography tool, was used to delineate the SLF and the cerebellar peduncles. This method provides a robust balance between the accuracy of manual delineation and the reliability/objectivity of atlas-based tracking approaches (Genc et al. 2020a; Wasserthal et al. 2018, 2019). TractSeg was applied to the study-specific population template to delineate the cerebellar peduncles (segmented into inferior, middle, and superior cerebellar peduncles as per the TractSeg library) and the SLF (segmented into SLF I, II, and III as per the TractSeg library). Figure 2 provides a visual representation of the delineated tracts.

### Statistical analyses

**Behavioural Analyses** We conducted a one-way repeated measures ANOVA with mean IES as the dependent variable and angle of rotation (0°, 45°, 90°, 135°, and 180°) as the within-subjects factor. This analysis was conducted to verify if participants were engaged in a mental rotation strategy during the task. To this end, analysis needed to demonstrate a linear increase in mean IES with increasing angular rotation (ter Horst et al. 2010). Handedness was not associated with HRT performance in this study (see supplementary materials).

**Fixel-wise Comparisons** In preparation for statistical analysis, participants’ fixel maps were cropped to only correspond to those fixels that were traversed by streamlines belonging to the cerebellar peduncles and the SLF. Using this approach, we created tract specific fixel maps for each participant corresponding to the cerebellar peduncles (segmented into inferior, middle, and superior cerebellar peduncles) and the SLF (segmented into SLF I, II, and III; as per Kamali et al. 2014; Schurr et al. 2020). For statistical analysis, white matter tracts were combined across hemispheres (e.g., we combined the left and right SLF I) to reduce the number of comparisons.

To assess the association between white matter fibre metrics (FD and FC) and MI performance, this study adopted fixel-based analysis (FBA; Dhollander et al. 2021). Fixel-based analysis is a state-of-the-art fibre specific analysis framework that enables the quantification of white matter fibre properties at the “fixel” level (within voxels). In doing so, FBA is well placed to ascribe individual differences in white matter organisation to specific white matter tracts and is less likely to be affected by interpretability issues that can arise in crossing fibre population when adopting traditional (voxel-wise) diffusion approaches such as diffusion tensor imaging. At the same time, FBA metrics are considered to provide greater biological interpretability relative to tensor derived voxel-wise diffusion metrics such as fractional anisotropy. This advance is highly valuable in developmental research where subtle differences in white matter organisation may be observed (Dhollander et al. 2021).

As per the recommended FBA pipeline (Tournier et al. 2019; Dhollander et al. 2021), fixel data were analysed using connectivity-based fixel enhancement (CFE) and permutation-based inference testing (Winkler et al. 2014). The CFE method provides a family-wise error (FWE) corrected *p*-value for each individual fixel within the tract of interest (Raffelt et al. 2015). In doing so, this analysis tests for regions of significant fixels along the SLF and the cerebellar peduncles that show an association between white matter fibre properties and MI performance. Age and sex were included as covariates (see supplementary Tables S1-S3 for analyses assessing the effects of covariates). Analyses involving FC were further adjusted for individual differences in intracranial volume (ICV; Smith et al., 2019), which was derived from structural T1-weighted images using FreeSurfer (Fischl 2012).

A significance value of *p*_*FWE*_ < 0.05 was set for all correlations. In tracts where significant associations were observed, mean FD and/or FC was calculated for each participant across all significant fixels (*p*_*FWE*_ < 0.05) to visualize the association between white matter fibre metrics and MI performance. Correlational analyses were conducted on the final sample of *N* = 18 participants for which both HRT and MRI data were available.

**Tract-based ROI Analysis** While fixel-wise analyses are well placed to identify localised effects (regions of significant fixels), they correct for many fixels (typically > 10,000 fixels) which can reduce their sensitivity (Bianco et al. 2023). To further explore the association between white matter organisation and MI, we computed mean FD and FC for each participant in each tract and then used Spearman’s correlations (rho) to examine the association between mean FD/FC in each tract and HRT performance. A non-parametric approach was chosen since RT data are typically positively skewed (Marmolejo-Ramos et al. 2015). Age and sex were included as covariates. Multiple comparisons across segments within each tract were adjusted for using a false discovery rate (FDR) of 0.05 (Benjamini and Hochberg 1995).

## Results

### HRT performance

Descriptive statistics for MI performance are presented in Table 1. As can be seen from this table, participants were more efficient (indicated by lower IES values) when responding to medially rotated stimuli compared to laterally rotated stimuli.

**Table 1:** Means and standard deviations (in brackets) for performance on the HRT (*N* = 18). Note. HRT = hand rotation task, RT = response time (ms), ACC = accuracy (proportion correct), IES = mean inverse efficiency (ms/proportion correct)

	Angular Rotation							Direction	
	0°	45°	90°	135°	180°		Medial		Lateral
RT	2131	2127	2490	2962	3220		2182		2855
	(643)	(618)	(783)	(1014)	(967)		(659)		(876)
ACC	0.85	0.85	0.85	0.83	0.75		0.92		0.77
	(0.16)	(0.20)	(0.16)	(0.23)	(0.26)		(0.12)		(0.28)
IES	2568	2710	2966	3838	5059		2446		4514
	(813)	(1245)	(909)	(1583)	(2628)		(864)		(2599)

**Fig. 3:**
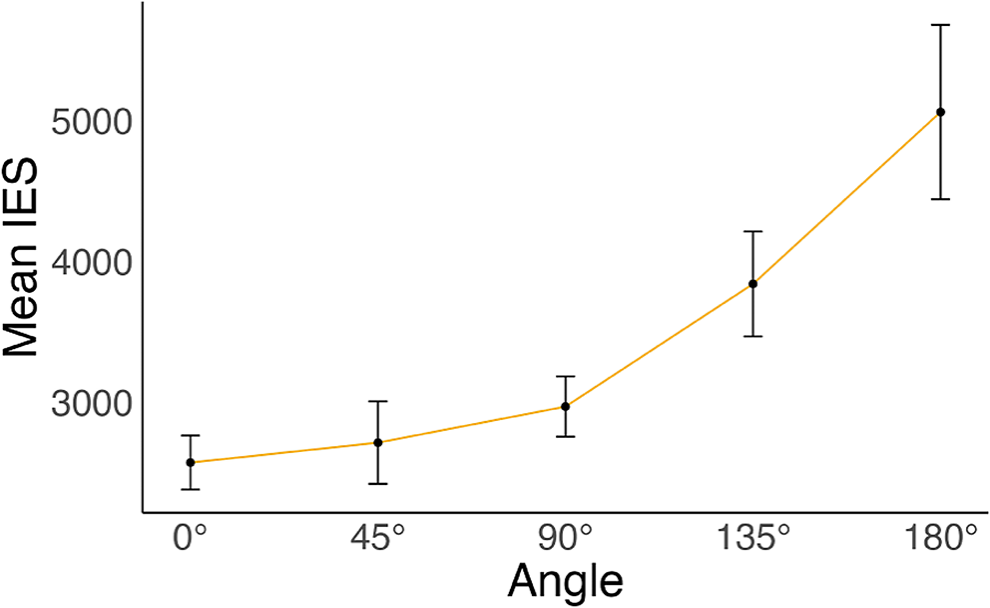
Results from the ANOVA showing average efficiency values (IES) across angular rotations for all participants. *Note*. IES = mean inverse efficiency (ms/proportion correct)

The repeated measures ANOVA comparing mean IES across angular rotations (0°, 45°, 90°, 135°, 180°) showed a significant linear trend (*B* = 1931.936, *SE* = 280.105, 95% CI [1372.995, 2490.878], *t*(68) = 6.90, *p* < .001) for angle *F*(4, 68) = 13.69, *p* < .001, *η*^*2*^_*p*_ *=* 0.45. See Fig. 3 for a visual representation of the results. For completeness of results, separate analyses for RT and accuracy are presented in the supplementary materials).

### Associations between white matter fixel-metrics and MI performance

#### Fixel-wise comparisons

**Fig. 4:**
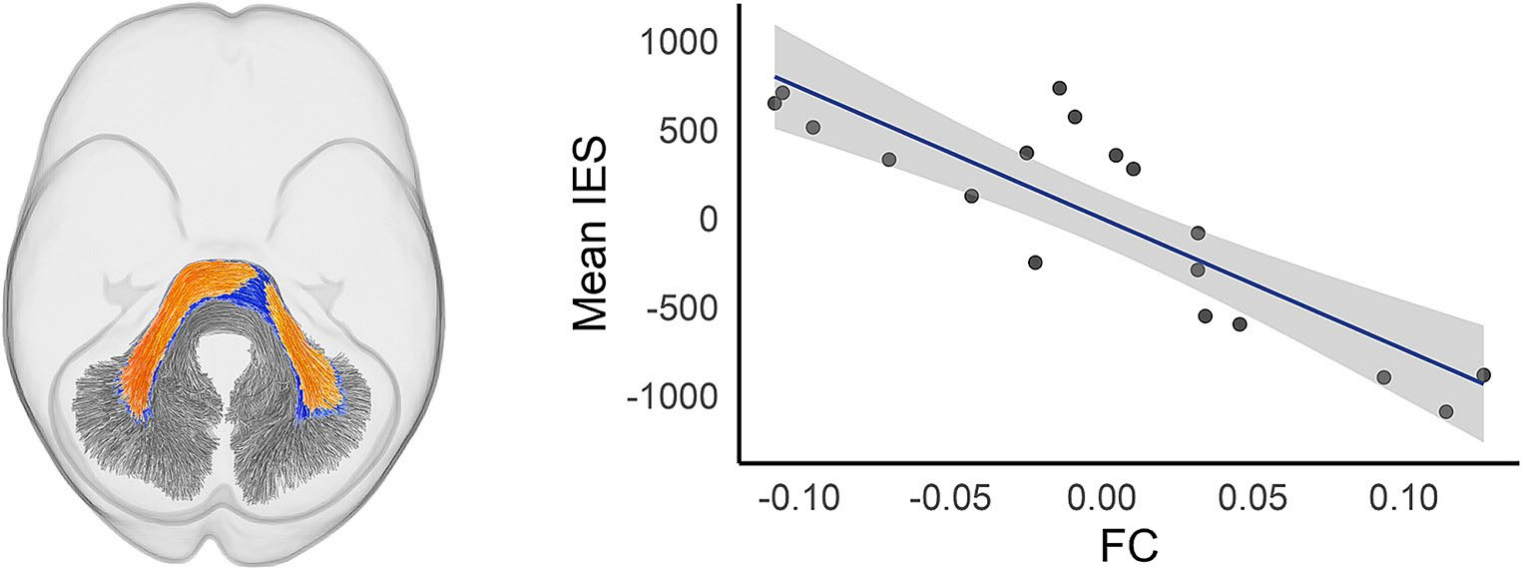
Streamline segments showing a significant negative correlation (*p*_*FWE*_ < 0.05) between mean IES and logFC within the middle cerebellar peduncle. To demonstrate the spatial extent of effects, results are presented using *p*_*FWE*_ < 0.05 (yellow region) and *p*_*FWE*_ < 0.10 (blue region). The scatterplot provides a visual representation of the association between mean logFC (averaged across the significant fixels form the CFE analysis) and mean IES

**White matter morphology (FC)** Fixel-wise comparisons showed significant negative associations between mean IES on the HRT and white matter morphology (logFC) in segments of the middle cerebellar peduncle (rho = − 0.88, *p*_*FWE*_ < 0.05). Results are presented in Fig. 4. Additional effects trending towards significance (*p*_*FWE*_ < 0.10) were observed in sections of the inferior cerebellar peduncles and the SLF I (see supplementary Figure S1).

**White matter microstructure (FD)** Fixel-wise comparisons did not reveal any significant associations between mean IES on the HRT and FD in any of our tracts of interest.

### Tract-based ROI analysis

**Fig. 5:**
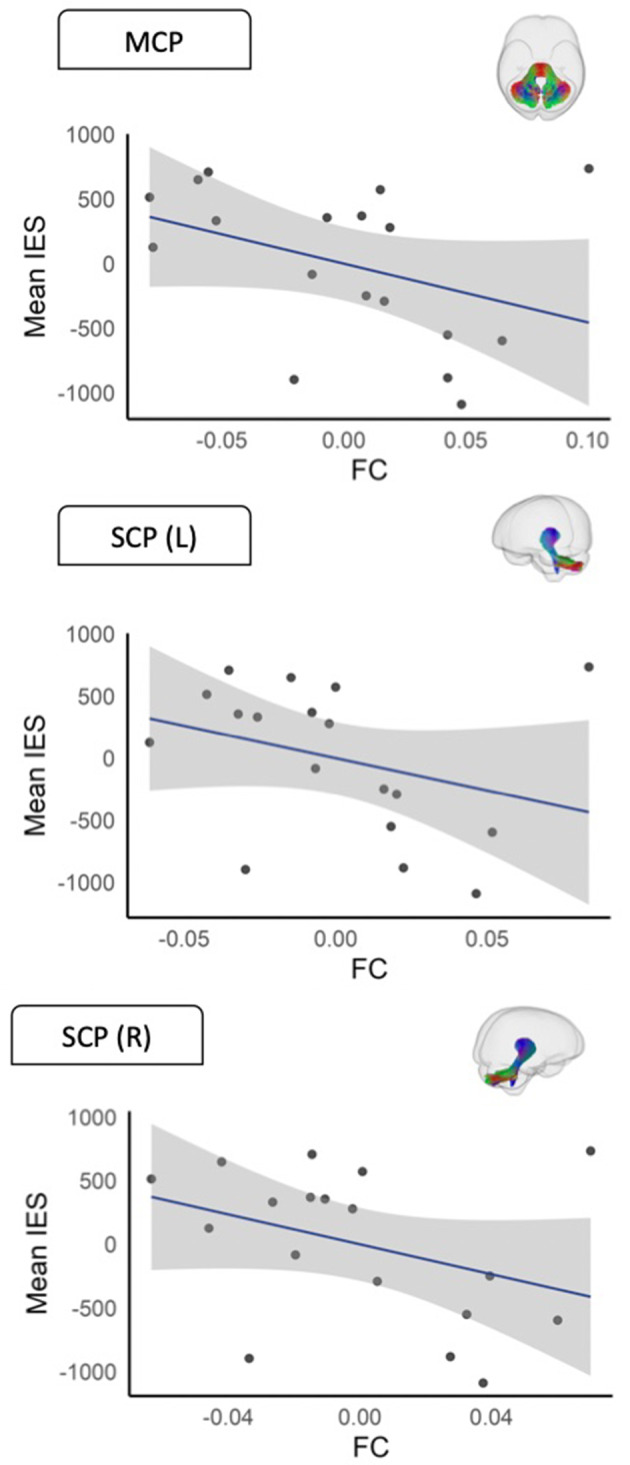
Scatterplots showing significant associations between IES and mean FC values (residualised). Covariates include age and sex, and intracranial volume. The shaded area represents standard error. FC = fibre bundle cross-section; L = left; R = right; MCP = middle cerebellar peduncle; SCP = superior cerebellar peduncle

**White matter morphology (FC)** Significant negative correlations were observed between mean IES on the HRT and mean FC of the middle cerebellar peduncle (rho = − 0.62, *p* = 0.013, *p*_*FDR*_ = 0.023), the left superior cerebellar peduncle (rho = − 0.63, *p* = 0.013, *p*_*FDR*_ = 0.023) and the right superior cerebellar peduncle (rho = − 0.62, *p* = 0.014, *p*_*FDR*_ = 0.023). We further observed an association for the left inferior cerebellar peduncle (rho = − 0.51, *p* = 0.052, *p*_*FDR*_ = 0.065) that fell just short of statistical significance. No significant association was observed for the right ICP, and no significant associations were observed for any of the SLF segments. Significant associations are shown in Fig. 5.

**Fig. 6:**
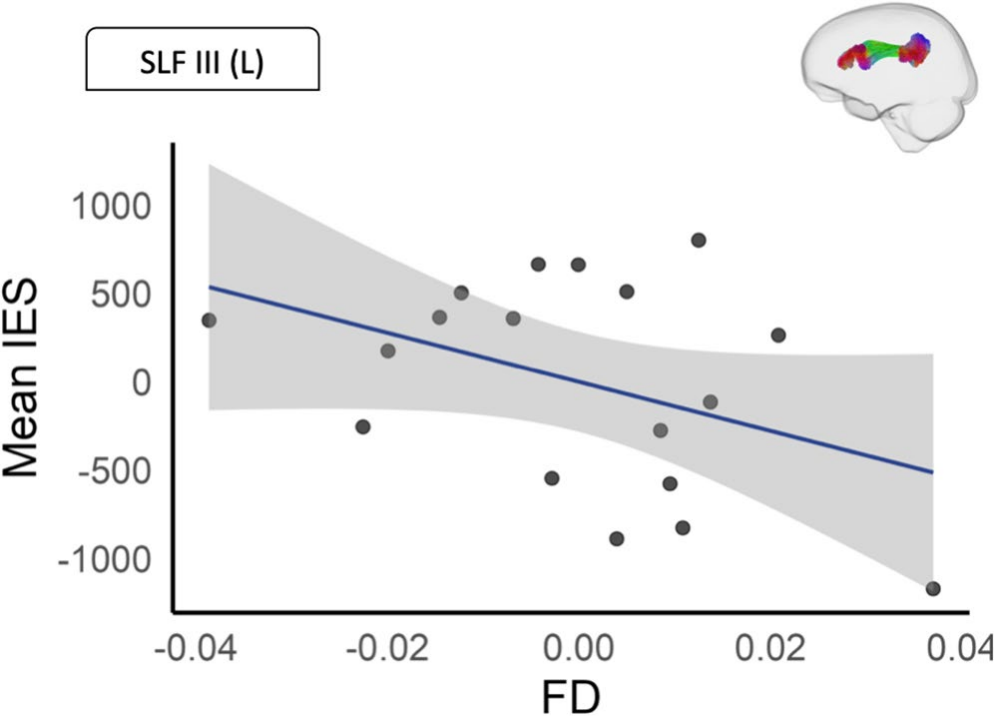
Scatterplot showing the association between IES and mean FD (residualised). Covariates include age and sex. The shaded area represents the standard error. FD = fibre density; L = left; SLF = superior longitudinal fasciculus

**White matter microstructure (FD)** Tract-ROI based analyses revealed a negative correlation between mean FD of the left SLF III and HRT performance (rho = − 0.56, *p* = 0.025, *p*_*FDR*_ = 0.150) which did not survive FDR correction. This association is shown in Fig. 6. No significant association were observed for the remaining SLF segments, and no significant associations were observed for any of the cerebellar peduncles.

## Discussion

To advance our understanding of the neurobiological basis of MI in childhood, this study examined the association between white matter fibre properties of sensorimotor tracts (cerebellar peduncles, SLF) and MI performance in children. Fixel-wise analyses revealed that higher FC (white matter morphology) in the middle cerebellar peduncle was associated with better MI performance. No significant associations were observed for white matter microstructural properties (FD). Tract-based ROI analyses examining mean FD and FC within each tract suggested that white matter organisation of the cerebellar peduncles and parts of the SLF were associated with MI performance. To our knowledge, this is the first study demonstrating the relevance of white matter organisation along sensorimotor tracts to MI performance in childhood. In doing so, this study builds on a strong body of fMRI evidence in adults suggesting overlap in those tracts responsible for overt and imagined movement.

### MI performance

Analysis of HRT performance indicated that participants were likely engaged in a MI strategy when performing the HRT. At a group level, analysis of HRT performance demonstrated a linear increase in IES (indicating lower performance) with increasing angular rotation. This pattern is consistent with previous studies adopting the HRT in childhood (Barhoun et al. 2019; Caeyenberghs et al. 2009) and supports the notion that participants adopted a mental rotation strategy to perform the task. Further, participants were more efficient when judging medially rotated hands compared to laterally rotated hands, suggesting that task performance reflected the biomechanical constraints of overt movement. Since this performance pattern is considered unique to MI (compared to visual imagery; de Lange et al. 2006; Spruijt et al. 2015a), we argue that HRT performance of children in our study likely reflected implementation of a MI strategy. Together, preliminary analyses thus demonstrated that HRT performance provided a valid indicator of the ability to generate and/or engage MI in the present study.

### Associations between White Matter Fibre Properties and MI Performance

**Cerebellar peduncles** As hypothesised, analysis demonstrated a negative association between HRT performance and white matter morphology (FC) of the middle cerebellar peduncle. This suggests that higher FC along the middle cerebellar peduncle was associated with better HRT performance. This effect was observed for both the fixel-wise analysis and the tract-based ROI analysis. Analyses further showed that higher average FC in the left and right superior cerebellar peduncles were associated with better MI performance.

Since FC is considered to reflect macrostructural properties of the fibre bundle that have been associated with individual differences in white matter connectivity (Raffelt et al. 2017), our results provide complementary evidence that the brain’s ability to relay information along the cerebellar peduncles may underlie MI performance in typically developing children. In doing so, our findings build on fMRI studies in adults (Hardwick et al. 2018; Hétu et al. 2013; Zapparoli et al. 2014) and highlight, for the first time, white matter correlates of implicit MI in typically developing children. Notably, considering that MI is thought to provide insight into the internal action representations that unconsciously precede and subserve movement (Gabbard 2009; Munzert et al. 2009), the results of this study are compatible with the view that white matter connectivity along the cerebellar peduncles may play a role in generating and/or engaging these internal movement representations.

Efferent connections from the cerebellum along the superior cerebellar peduncles are considered to play a role during forward modelling (Welniarz et al. 2021), while the middle cerebellar peduncle has been implicated in a range of motor functions that are considered to place demands on internal movement representations, including balance (Odom et al. 2021) and manual dexterity (Thomas et al. 2017). In the context of this work, our findings could indicate that white matter connectivity along these tracts may support important cognitive and motor functions in childhood. However, without experimental data assessing these functions directly, it is difficult to draw firm conclusions from our results.

No significant associations were found between microstructural properties (FD) of the cerebellar peduncles and MI performance. A possible explanation for these results is that differences in white matter morphological properties (rather than microstructural properties) underlie MI performance in childhood. However, given that FC can be sensitive to different neurobiological properties, including myelination and extra-cellular space (Genc et al. 2020b), future studies investigating the specific contribution of these properties will provide additional insight into the neurobiological mechanisms that underlie childhood MI.

**Superior longitudinal fasciculus** Results from the tract-based ROI analysis suggested that higher mean FD (white matter microstructure) in the left SLF III was associated with improved MI performance. FD represents a measure of the density or number of axons within a voxel, such that higher FD could reflect a tightly packed fibre bundle or a greater number of axons within a voxel (Raffelt et al. 2017). Given that higher mean FD in the left SLF III was associated with improved MI performance, our results are compatible with the view that an increased ability to relay information along the SLF III may facilitate MI processes in childhood. This finding builds on fMRI evidence from adult studies which showed that cortical regions connected by the SLF III (e.g., inferior parietal lobe, inferior frontal gyrus; Hardwick et al. 2018; Hétu et al. 2013) show an increased BOLD response during MI.

Functionally, the SLF III has been associated with temporal aspects of movement (Budisavljevic et al. 2017) and higher order cognitive functions including working memory (Parlatini et al. 2017). Since MI processes are largely thought to take place in working memory (Gabbard et al. 2013; Schott 2012), our finding raises the possibility that white matter connectivity along the SLF III may support MI processes that place demands on the working memory system (e.g., monitoring internal movement representations, inhibition of movement execution). However, in the absence of working memory measures, and considering that the observed association was no longer significant after adjusting for multiple comparisons, this notion must be interpreted with caution.

No significant associations were found between white matter organisation of the SLF I and II and MI performance. This was unexpected since both the SLF I and II connect several cortical regions commonly implicated during MI (Hardwick et al. 2018; Hétu et al. 2013) and given the broader role of the SLF in sensorimotor processes (Urger et al. 2015). However, rather than suggesting the genuine absence of an effect, it is possible that the hypothesized association may have been more subtle than anticipated. In support of this view, our fixel-wise analysis showed a non-significant trend (*p*_*FWE*_ < 0.10) towards a negative association between white matter morphology (FC) in the SLF I and MI performance. Alternatively, a possible explanation for this finding could be that the SLF is not yet fully developed in childhood and that the association between MI performance and structural properties of the SLF is therefore reduced. While this interpretation is consistent with protracted maturation of the SLF into adolescence (Amemiya et al. 2021; Lebel and Deoni 2018), longitudinal data mapping the development of the SLF and MI performance into adolescence is needed to investigate this premise further.

### Implications, limitations, and future directions

To our knowledge, this has been the first study adopting FBA to examine the association between white matter fibre properties of sensorimotor tracts and MI performance in childhood. Results from this study add to our understanding of the neurobiological basis of MI in childhood and extend previous fMRI work in adults reporting on task-based activation during the HRT. Notably, while a strong body of evidence suggests overlap in the sensorimotor neural systems that support overt and imagined movement in adults (Kilteni et al. 2018), little is known about the sensorimotor neural systems that support MI in children. Our results provide new evidence in relation to this knowledge gap, suggesting that the ability to relay information efficiently along sensorimotor white matter pathways may be associated with MI in childhood.

From a theoretical perspective, our results are compatible with well-established computational models of motor control. These models suggest that the cerebellum plays an important role in the generation of internal representations of movement, which are essential to a mature and flexible motor system (Franklin & Wolpert, 2011; Ishikawa et al. 2016). Given that MI is considered to provide a window into the internal representations of action that subserve movement (Gabbard 2009; Munzert et al. 2009), and since MI performance was robustly associated with white matter organisation of the middle cerebellar peduncle in this study, our findings highlight cerebellar white matter as a possible neural marker of the ability to generate and/or engage internal action representations in childhood. This information is valuable as it enhances our understanding of typical brain and cognitive development and provides early evidence for the relevance of white matter sensorimotor pathways to internal action representations.

Despite adopting a rigorous methodological approach, including a well-validated measure of implicit MI, a novel and fibre specific analysis framework and a state-of-the art tractography technique, this study is not without limitations. While this has been the first study to examine the contribution of the cerebellar peduncles and the SLF to MI performance in childhood, our sample size was modest. Future studies should look to replicate the present findings in a larger cohort and consider additional white matter pathways that could be implicated during MI. This should also present a valuable opportunity to consider possible white matter correlates of MI performance in populations where MI has been shown to develop atypically, as is the case in children with Developmental Coordination Disorder (e.g., Barhoun et al. 2019) or Attention-Deficit/Hyperactivity Disorder (Williams et al. 2013).

An additional white matter tract that could be implicated during MI is the corticospinal tract (CST). The CST is thought to receive most of its output from the primary motor cortex (M1), the final relay point for voluntary control signals before transmission to peripheral muscles via the spinal cord (Welniarz et al. 2017). We opted to not include the CST in the present study given that the involvement of the primary motor cortex in MI is heavily debated. Indeed, while suprathreshold muscle activity is observed during MI, by definition, overt movement is suppressed. Hence, the motor command that would ordinarily descend the CST during overt movement should be largely inhibited during MI (Grosprêtre et al. 2016).

Thus, involvement of the CST during MI would be expected to be reduced, or negligible, relative to the SLF and the cerebellar peduncles which support communication between sensorimotor grey matter regions that are commonly implicated during MI. Given our modest sample size, we were unfortunately unable to include tracts of interest for which there was not a strong case for involvement in MI. Still, given the motoric nature of MI, future studies should consider additional white matter pathways that could be implicated during MI, including the CST.

## Conclusion

To our knowledge, this has been the first study to demonstrate that white matter organisation of the cerebellar peduncles and segments of the SLF are associated with individual differences in MI performance in childhood. These findings advance our understanding of the neurobiological systems that underlie MI performance and highlight the possible relevance of white matter sensorimotor pathways to internal movement representations in childhood. Future research should look to replicate our findings in a larger cohort and consider possible white matter correlates of MI performance in populations where MI has been shown to develop atypically.

## Electronic supplementary material

Below is the link to the electronic supplementary material.

Supplementary Material 1

## Data Availability

The conditions of our ethics approval do not permit public archiving of anonymised study data. Readers seeking access to the data should contact the lead author or the relevant local ethics committee. Access will be granted to named individuals in accordance with ethical procedures governing the reuse of clinical data, including completion of a formal data sharing agreement and approval of the local ethics committee. Code used for data processing and analysis is publicly available and provided on the MRtrix3 (https://www.mrtrix.org) and FreeSurfer (https://surfer.nmr.mgh.harvard.edu) websites. Legal copyright restrictions prevent public archiving of the various assessment instruments used in this study, which can be obtained from the copyright holders in the cited references.
