# Supplementary material for: White matter organisation of sensorimotor tracts is associated with motor imagery in childhood
Source: Brain Struct Funct. 2024 Jun 25;229(7):1591–603. doi: 10.1007/s00429-024-02813-4 (PMC11374871; doi:10.1007/s00429-024-02813-4)
Supplement: Supplementary file 1 — Supplementary Material 1 [file 429_2024_2813_MOESM1_ESM.docx]

**Supplementary Material**

**White matter organisation of sensorimotor tracts is associated with motor imagery in childhood.**

**Mugdha Mukherjee^a^ *, Christian Hyde^a^, Pamela Barhoun^a^, Kaila M. Bianco^a^, Mervyn Singh^a^, Jessica Waugh^a^, Timothy J. Silk^a^, Jarrad A.G. Lum^a^, Karen Caeyenberghs^a^, Jacqueline Williams^b^, Peter G. Enticott^a^, Ian Fuelscher^a^**

**Author affiliations**

^a^School of Psychology, Deakin University, Geelong, Victoria, Australia

^b^Institute for Health and Sport, Victoria University, Melbourne, Victoria, Australia

*** Corresponding author: mmukherj@deakin.edu.au**

**Journal: Brain Structure and Function**

**Effect of Handedness**

The sample included four left-handed participants. To examine the possible impact of handedness on hand rotation task (HRT) performance, we compared left- and right-handed participants on HRT performance after covarying for age. ANCOVA showed no significant differences in IES between the groups, *F*(1, 15) = 0.43, *p* = 0.524, η^2^_p_ = .03. Thus, it is unlikely that differences in handedness impacted HRT performance in the present study.

**Effect of Age**

To investigate the effects of age on white matter organisation of the superior longitudinal fasciculus (SLF) and the cerebellar peduncles, we examined the association between age and fibre metrics (averaged across all fixels) for each tract segment separately. Analyses are presented in Table S1. Although significant associations were only observed for some tract segments, age was still included as a covariate as it is commonly considered to be associated with white matter fixel metrics (FD and FC) in childhood (Genc et al., 2018).

| **Tract of interest** | **FD** | | | | **FC** | | | |
| --- | --- | --- | --- | --- | --- | --- | --- | --- |
|  | ***M*** | ***SD*** | ***r*** | ***p*** | ***M*** | ***SD*** | ***r*** | ***p*** |
| ICP left | .34 | .03 | -.08 | .759 | 1.06 | .05 | .12 | .640 |
| ICP right | .29 | .02 | -.11 | .650 | 1.05 | .05 | .12 | .622 |
| MCP | .36 | .03 | -.21 | .406 | 1.07 | .05 | .07 | .795 |
| SCP left | .45 | .02 | -.03 | .906 | 1.02 | .04 | -.04 | .876 |
| SCP right | .43 | .02 | -.10 | .691 | 1.02 | .04 | .05 | .845 |
| SLF I left | .33 | .02 | .64 | .004 | 1.09 | .06 | -.08 | .764 |
| SLF I right | .37 | .02 | .55 | .019 | 1.08 | .06 | -.13 | .610 |
| SLF II left | .32 | .02 | .37 | .132 | 1.10 | .09 | -.07 | .773 |
| SLF II right | .32 | .02 | .29 | .235 | 1.10 | .08 | -.12 | .623 |
| SLF III left | .31 | .02 | .36 | .144 | 1.09 | .08 | .11 | .650 |
| SLF III right | .31 | .01 | .35 | .160 | 1.10 | .08 | -.08 | .767 |

**Supplementary Table S1.** FD = fibre density; FC = fibre bundle cross-section; *M* = mean; *SD* = standard deviation; *r* = Pearson’s correlation coefficient; ICP = inferior cerebellar peduncle; MCP = middle cerebellar peduncle; SCP = superior cerebellar peduncle; SLF = superior longitudinal fasciculus

**Effect of Sex**

To investigate the possible effect of sex on white matter organisation of the SLF and the cerebellar peduncles, we compared fibre metrics (averaged across all fixels) between males and females for each tract. Analyses are presented in Table S2. Although significant differences were only observed for some tract segments, sex was still included as a covariate since sex differences are commonly reported for white matter fixel metrics (FD and FC) in childhood (Genc et al., 2018).

| **Tract of interest** | **FD** | |  | **FC** | |
| --- | --- | --- | --- | --- | --- |
|  | ***t*** | ***p*** |  | ***t*** | ***p*** |
| ICP left | 0.07 | .947 |  | 0.23 | .821 |
| ICP right | -0.05 | .960 |  | 0.83 | .419 |
| MCP | 0.59 | .565 |  | 1.08 | .297 |
| SCP left | -0.07 | .949 |  | 0.95 | .358 |
| SCP right | -.018 | .860 |  | 1.08 | .298 |
| SLF I left | -1.40 | .181 |  | 1.88 | .078 |
| SLF I right | -0.98 | .342 |  | 1.85 | .082 |
| SLF II left | -0.67 | .513 |  | 1.60 | .130 |
| SLF II right | -0.31 | .761 |  | 1.76 | .098 |
| SLF III left | -1.35 | .196 |  | 2.53 | .022 |
| SLF III right | -0.86 | .404 |  | 3.15 | .006 |

**Supplementary Table S2.** FD = fibre density; FC = fibre bundle cross-section; ICP = inferior cerebellar peduncle; MCP = middle cerebellar peduncle; SCP = superior cerebellar peduncle; SLF = superior longitudinal fasciculus

**Effect of intracranial volume (ICV)**

Individual differences in intracranial volume (ICV) have been adjusted for in some studies investigating FC in childhood (see Smith et al., 2019) but not others (Genc et al., 2018; Honnedevasthana Arun et al., 2021). To examine the possible effect of ICV in the present study, we examined the association between ICV and FC (averaged across all fixels) for each tract segment. Analyses are presented in Table S3. Since ICV was significantly associated with FC in the SLF, we re-ran our analyses examining the association between FC and MI performance with the inclusion of ICV as an additional covariate. Results suggested that the inclusion of ICV as an additional covariate did not change the overall interpretation of findings. Accordingly, ICV was not included as an additional covariate in or main analyses.

| **Tract of interest** | **Correlation between FC and ICV** | |
| --- | --- | --- |
|  | ***r*** | ***p*** |
| ICP left | -.02 | .932 |
| ICP right | .05 | .855 |
| MCP | .21 | .399 |
| SCP left | .37 | .126 |
| SCP right | .40 | .099 |
| SLF I left | .73 | <.001 |
| SLF I right | .71 | <.001 |
| SLF II left | .76 | <.001 |
| SLF II right | .78 | <.001 |
| SLF III left | .76 | <.001 |
| SLF III right | .74 | <.001 |

**Supplementary Table S3.** FC = fibre bundle cross-section; ICV = intracranial volume; ICP = inferior cerebellar peduncle; MCP = middle cerebellar peduncle; SCP = superior cerebellar peduncle; SLF = superior longitudinal fasciculus

**Motion Estimates**

To mitigate possible effects of head motion in this study, between volume motion correction was performed using the methods outlined by Andersson & Sotiropoulos (2016). Motion estimates were derived from the available eddy QC output generated as part of the recommended FBA pipeline (Dhollander et al., 2021; Tournier et al., 2019).

|  | ***n* = 22** | |
| --- | --- | --- |
|  | ***M*** | ***SD*** |
| Average absolute motion (mm) | 0.85 | 0.69 |
| Average relative motion (mm) | 0.29 | 0.29 |
| Average x translation (mm) | -0.05 | 0.24 |
| Average y translation (mm) | -0.03 | 0.37 |
| Average z translation (mm) | -0.04 | 0.73 |
| Average x rotation (deg) | -.03 | .45 |
| Average y rotation (deg) | .10 | .21 |
| Average z rotation (deg) | .04 | .18 |

**Supplementary Table S4.** Average motion parameters for participants (*N* = 22). The parameters for these corrections are presented here. *M* = mean; *SD* = standard deviation.

**Fixel-wise (CFE) Analysis**

Besides the analyses reported in the main manuscript, we observed non-significant trends (p_FWE_ < .10) for the association between fibre metrics (FC) and MI performance in sections of the inferior cerebellar peduncles and the SLF I. These trends are shown in Figure S1.

**
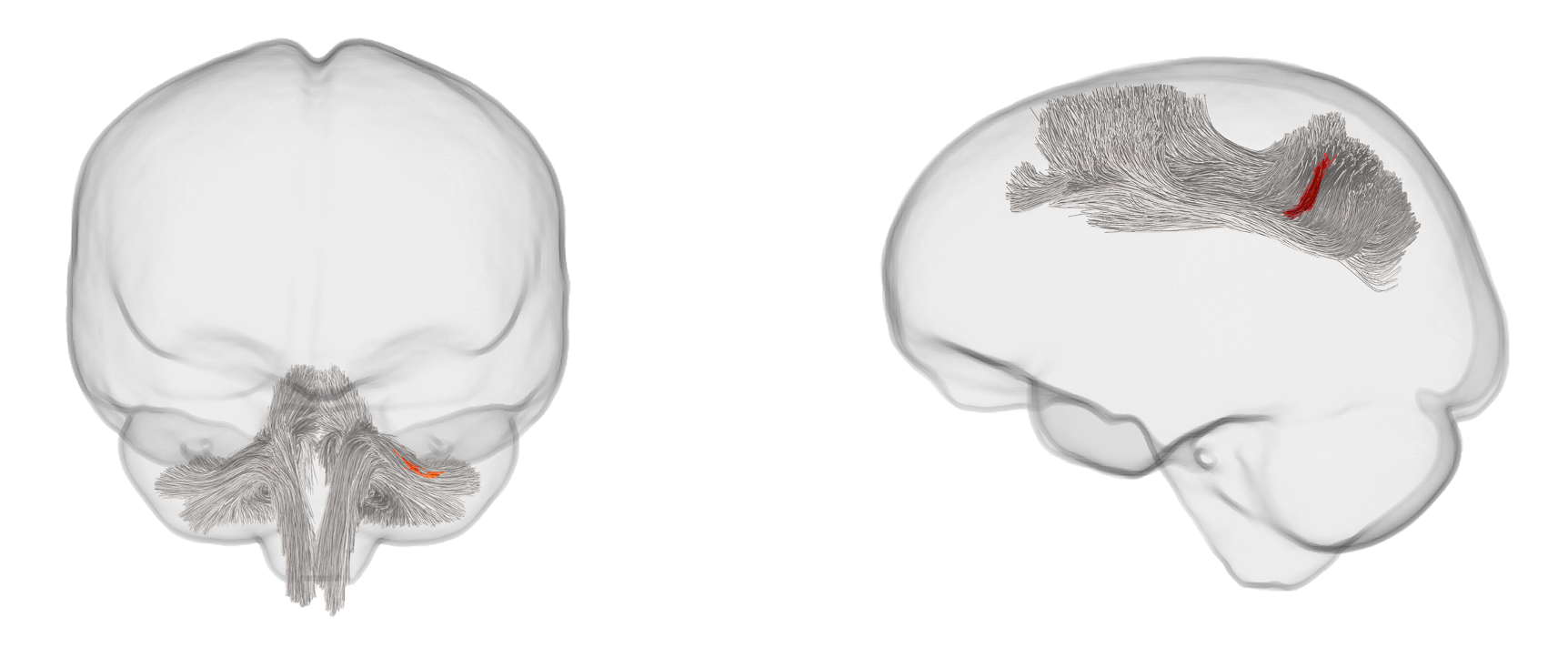
**

**Fig S1** Streamline segments showing trends towards significant negative correlations (p_FWE_ < .10) between mean IES and logFC within the inferior cerebellar peduncles (presented in orange) and the SLF I (presented in red)

**MI Performance**

Studies examining HRT performance often report separate analyses for response time (RT) and accuracy. For completeness of result, these analyses are presented below. As can be seen from these analyses, we observed comparable effects (linear trends) for mean IES (reported in the main manuscript), RT, and accuracy.

A one-way repeated measures ANOVA comparing response times across angular rotations (0°, 45°, 90°, 135°, 180°) showed a significant linear trend (*B* = 952.985, *SE* = 107.111, 95% CI [739.249, 1166.721], *t*(68) = 8.90, *p* < .001) for angle *F*(4, 68) = 21.15, *p* <.001, η^2^_p_ = .55. See Figure S2 for a visual representation of the results.

A one-way repeated measures ANOVA also comparing accuracy across angular rotations (0°, 45°, 90°, 135°, 180°) showed a significant linear trend (*B* = -.070, *SE* = .025, 95% CI [-.121, -.020], *t*(68) = -2.767, *p* = .007) for angle *F*(4, 68) = 2.98, *p* = .025, η^2^_p_ = .15. See Figure S3 for a visual representation of the results.


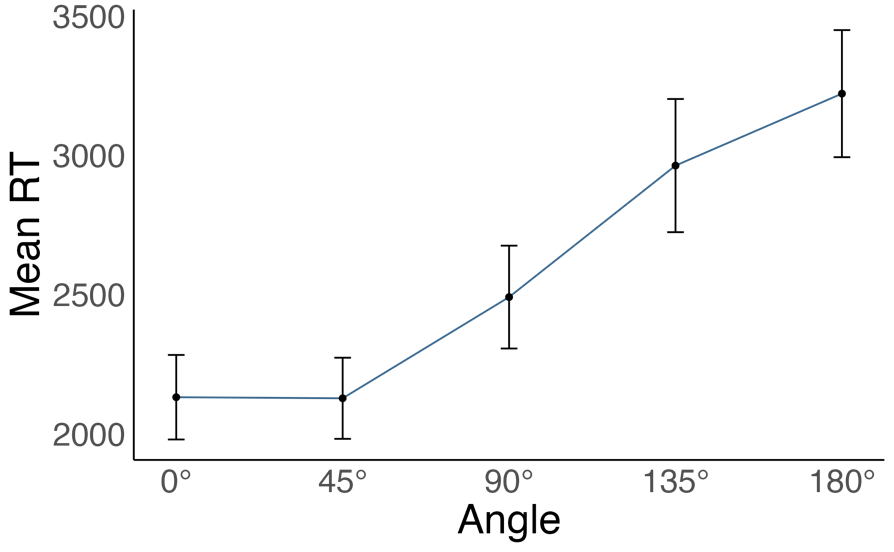


**Fig S2** Results from the ANOVA showing average response time values across angular rotations for all participants. *Note*. RT = response time (in ms)


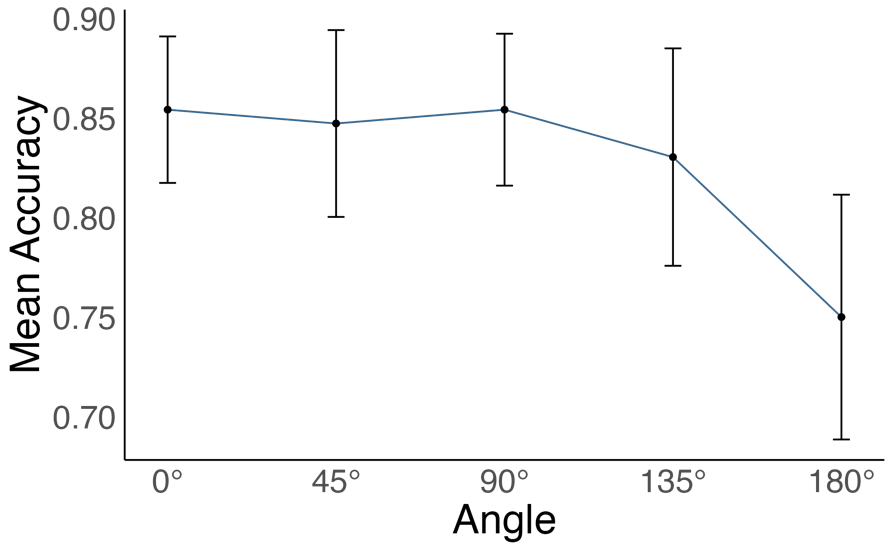


**Fig S3** Results from the ANOVA showing average accuracy values across angular rotations for all participants

**References**

Andersson, J. L. R., & Sotiropoulos, S. N. (2016). An integrated approach to correction for off-resonance effects and subject movement in diffusion MR imaging. *NeuroImage*, *125*, 1063–1078. https://doi.org/10.1016/j.neuroimage.2015.10.019

Dhollander, T., Clemente, A., Singh, M., Boonstra, F., Civier, O., Duque, J. D., Egorova, N., Enticott, P., Fuelscher, I., Gajamange, S., Genc, S., Gottlieb, E., Hyde, C., Imms, P., Kelly, C., Kirkovski, M., Kolbe, S., Liang, X., Malhotra, A., … Caeyenberghs, K. (2021). Fixel-based Analysis of Diffusion MRI: Methods, Applications, Challenges and Opportunities. *NeuroImage*, *241*, 118417. https://doi.org/10.1016/j.neuroimage.2021.118417

Genc, S., Smith, R. E., Malpas, C. B., Anderson, V., Nicholson, J. M., Efron, D., Sciberras, E., Seal, M. L., & Silk, T. J. (2018). Development of white matter fibre density and morphology over childhood: A longitudinal fixel-based analysis. *NeuroImage*, *183*, 666–676. https://doi.org/10.1016/j.neuroimage.2018.08.043

Honnedevasthana Arun, A., Connelly, A., Smith, R. E., & Calamante, F. (2021). Characterisation of white matter asymmetries in the healthy human brain using diffusion MRI fixel-based analysis. *NeuroImage*, *225*, 117505. https://doi.org/10.1016/j.neuroimage.2020.117505

Smith, R., Dhollander, T., & Connelly, A. (2019). *On the regression of intracranial volume in Fixel-Based Analysis*.

Tournier, J.-D., Smith, R., Raffelt, D., Tabbara, R., Dhollander, T., Pietsch, M., Christiaens, D., Jeurissen, B., Yeh, C.-H., & Connelly, A. (2019). MRtrix3: A fast, flexible and open software framework for medical image processing and visualisation. *NeuroImage*, *202*, 116137. https://doi.org/10.1016/j.neuroimage.2019.116137
